# Substrate-specific mitochondrial bioenergetics and metabolic flexibility in visceral adipose tissue across obesity-related glycemic states

**DOI:** 10.1080/21623945.2026.2751414

**Published:** 2026-11-11

**Authors:** Daniel A. Mendonça, Raquel L. Bernardino, Marcelo Topete, Sara Andrade, Marta Guimarães, Ana M. Pereira, Sofia B. Oliveira, Madalena M. Costa, Mário Nora, Mariana P. Monteiro, Sofia S. Pereira

**Affiliations:** a Unit for Multidisciplinary Research in Biomedicine, School of Medicine and Biomedical Sciences, University of Porto, Porto, Portugal; b ITR-Laboratory of Integrative and Translation Research in Population Health, Porto, Portugal; c Department of General Surgery, Hospital São Sebastião, Unidade Local de Saúde de Entre o Douro e Vouga, Santa Maria da Feira, Portugal; d Hospital da Luz Arrábida, Vila Nova de Gaia, Portugal

**Keywords:** Bioenergetics, obesity, prediabetes, type 2 diabetes, visceral adipose tissue

## Abstract

Obesity is characterized by the expansion of metabolically dysfunctional visceral adipose tissue (VAT) and chronic low-grade inflammation, which contribute to the development of insulin resistance. However, to what extent VAT bioenergetics and substrate preferences differ across distinct obesity-related glycaemic status profiles remains poorly understood. The present study aimed to assess substrate utilization and metabolic flexibility of VAT from individuals with obesity across different glycaemic statuses, namely euglycemia, prediabetes, and type 2 diabetes (T2D). VAT from individuals with obesity with euglycemia (*n* = 11), prediabetes (*n* = 12) and T2D (*n* = 6) was harvested during bariatric surgery. VAT bioenergetics and mitochondrial substrate preferences under the influence of pyruvate, glutamine and fatty acids metabolism inhibitors, were assessed using the Seahorse Analyser. VAT expression of *OB, GLS1, MPC1, CPT1a, HK2, PDHA1, RPII, β-actin* genes was quantified by qRT-PCR. VAT basal mitochondrial respiration was similar across glycaemic groups. In VAT from euglycemic individuals, pyruvate inhibition decreased spare respiratory capacity. In contrast, in VAT from individuals with prediabetes and T2D, ATP-linked respiration increased when glutamine was the sole substrate available, which in individuals with prediabetes, was accompanied by an increase of non-mitochondrial oxygen consumption. There were no differences in the expression of genes related to energy metabolism across groups. These exploratory findings suggest that substrate-dependent VAT respiratory responses may vary across obesity-related glycaemic states despite the absence of detectable basal respiratory differences. The response observed under glutamine-only conditions raises the possibility of compensatory metabolic adaptations in prediabetes, although their mechanisms and potential alteration in T2D remain to be established.

## Introduction

Adipose tissue (AT) serves as a central regulator of overall energy homoeostasis, functioning not only as an energy storage depot but also as a metabolic endocrine organ [1]. The capacity to appropriately store and mobilize energy is critical for metabolic health, a function that is compromised in obesity, where dysregulation of energy mechanisms contributes to the development of obesity‑associated metabolic disorders [2].

Different adipose tissue depots display distinct metabolic and inflammatory characteristics, with visceral adipose tissue (VAT) being particularly implicated in metabolic dysfunction [3]. Unlike subcutaneous AT (SAT), VAT exhibits enhanced free fatty acid release and is highly infiltrated by pro-inflammatory macrophages, which sustain a state of chronic low-grade inflammation [4]. These pathological features impact insulin resistance and metabolic dysfunction through multiple mechanisms, including altered adipokine secretion and the activation of pro-inflammatory signalling cascades [5].

In obesity, mitochondrial biogenesis and functions related to lipogenesis and lipolysis, oxidative phosphorylation, free fatty acids (FFAs) oxidation, and branched-chain amino acids (BCAAs) catabolism are commonly impaired [6,7]. These alterations contribute to metabolic inflexibility, defective adipogenesis, and reduced AT expandability, which limit the appropriate storage of excess energy in adipose depots [8]. As a result, FFAs are redistributed, leading to ectopic fat accumulation in non-AT organs, such as the liver, pancreas, skeletal muscle, and heart, where they may induce lipotoxicity [8,9]. Impaired adipose tissue expandability promotes adipocyte hypertrophy and leads to tissue hypoxia, fostering a microenvironment that favours immune cell recruitment and activation. This pro-inflammatory macrophage infiltration enhances local production of cytokines that exacerbate insulin resistance by disrupting insulin signalling pathways, in part through increased generation of reactive oxygen species (ROS) [10,11]. The resulting oxidative stress further exacerbates the disruption of insulin action, thereby contributing to the progression of insulin resistance, which, along with the systemic pro-inflammatory and lipotoxic state, contributes to impairing β-cell function and to the development of T2D [10,12].

Prediabetes is characterized by high blood glucose levels, yet below the threshold required for T2D diagnosis. Being a dysglycemic state, prediabetes heralds the high risk of developing cardiovascular diseases and progression to T2D [13]. More importantly, this condition provides a window of opportunity for early intervention, since many individuals with prediabetes can either maintain a stable glucose status or even revert to euglycemia with lifestyle, pharmacological, or surgical interventions, as appropriate [14,15].

There is evidence supporting that mitochondrial dysfunction plays a major role in prediabetes and T2D development [16,17]. In particular, a recent study showed that AT from individuals with obesity and prediabetes preferentially utilized pyruvate, malate, glutamate, and/or fatty acids during oxidative phosphorylation, whereas AT from individuals with T2D primarily relied on succinate oxidation [17]. However, several key aspects remain incompletely characterized. In particular, substrate preferences and metabolic flexibility in VAT across obesity-related glycaemic states have not been systematically evaluated through sequential and selective modulation of individual substrates, such as pyruvate, glutamine, and fatty acids. Understanding how substrate utilization patterns in VAT shift across glycaemic statuses may provide critical mechanistic insights into VAT-specific metabolic dysfunction and help to identify targets for interventions to restore metabolic balance.

## Materials and methods

### Participant recruitment

Individuals with obesity undergoing laparoscopic surgery for the treatment of obesity or obesity-related disorders who provided written informed consent and signed an informed consent declaration were recruited for this study (*n* = 29). The study was approved by the ethical committee of Centro Hospitalar de Entre Douro e Vouga (ref. CA 0830/16-Ot) and Hospital da Luz Arrábida (ref. 1/202). All procedures performed in studies involving human participants were in accordance with the ethical standards of the institutional committee and with the principles stated in the Declaration of Helsinki.

According to pre-established inclusion and exclusion criteria, all individuals included were females, within classes 2 to 5 of obesity and euglycemia, prediabetes or T2D. Participants were allocated into three different groups according to the glycaemic status: euglycemia (*n* = 11), prediabetes (*n* = 12) or T2D (*n* = 6). No formal a priori sample size calculation was performed. The sample size was determined by the availability of eligible participants and surgical VAT samples during the recruitment period.

VAT was harvested during the bariatric surgical intervention and immediately transported in Dulbecco’s Modified Eagle Medium (DMEM), supplemented with PenStrep (ready-to-use antibiotic mix of penicillin and streptomycin) and Foetal Bovine Serum (FBS), from the surgical theatre to the laboratory for analysis. The harvested VAT was divided into 21 fragments of approximately 5 mg for bioenergetics analysis. The remaining tissue was then stored in a −80°C freezer for later total RNA extraction.

### Bioenergetic analysis

#### Oxygen consumption rate evaluation

To assess the VAT bioenergetic profile, we employed the Seahorse XFe24 Analyser, which evaluates the cells or tissues’ oxygen consumption rate (OCR) in a multi-well plate format. On the day before the assay, the sensor cartridge was hydrated using a commercial calibration buffer (XF Calibrant pH 7.4, Agilent Technologies, Santa Clara, CA, USA) and incubated at 37°C in the absence of CO_2_. On the day of the experiment, each VAT fragment was positioned at the bottom of the well using capture screens and pre-incubated in commercial assay medium (Seahorse XF DMEM Medium, pH 7.4, 103,575-100, Agilent Technologies) for approximately 30 minutes at 37°C in a CO_2_-free incubator to allow for stabilization. Following this, the medium was removed and replaced with Seahorse assay medium enriched with 0.01 M glucose (103577–100, Agilent Technologies), 2 mM glutamine (103579–100, Agilent Technologies), 0.17 mM sodium palmitate (P9767-5 G, Sigma-Aldrich, St. Louis, MO, USA) and 0.5 mM L-carnitine (C0283-1 G, Sigma-Aldrich), as previously described [18].

The electron transport chain (ETC) modulators and inhibitors of the metabolic pathways were pre-loaded into the cartridge and injected successively into the VAT wells after an initial equilibration period of 52 minutes. The A ports included various metabolic inhibitors: the mitochondrial pyruvate carrier (MPC1) blocker of UK5099 at a final concentration of 4 µM (5.04817.0001, EMD Millipore Corporation, USA); the GLS1 inhibitor BPTES (Bis-2-(5-phenylacetamido-1,3,4-thiadiazol-2-yl)ethyl sulphide) at 6 µM (SML0601, Sigma Aldrich); and Etomoxir, an inhibitor of carnitine palmitoyltransferase-1 (CPT1a), at 8 µM (236020, EMD Millipore Corporation). These inhibitors were administered separately or in combination of two. For the control group, a medium with 0.2% dimethyl sulphoxide (DMSO) (D2650, Sigma Aldrich), which is the vehicle for the inhibitors, was used. All experimental conditions were conducted in triplicate. Ports B, C and D were loaded with modulators of the ETC: port B contained oligomycin, an ATP synthase inhibitor, at a final concentration of 12.5 µg/mL (O4876, Sigma Aldrich); port C was filled with carbonyl cyanide-p-trifluoromethoxyphenylhydrazone (FCCP), a mitochondrial oxidative phosphorylation uncoupler, at 8 µM (C2920, Sigma Aldrich); and port D contained a mixture of Rotenone and Antimycin A, inhibitors of mitochondrial complexes I and III, respectively, at final concentrations of 2 µM (R8875, Sigma Aldrich) and 4 µM (A8674, Sigma Aldrich). VAT’s oxygen consumption rate was measured for 6 hours and 40 minutes by the Seahorse XFe24 Analyser. At the end of the assay, the VAT fragments were recollected from the plate and frozen at −20°C for later protein extraction. The results were exported and analysed using the Seahorse Wave 2.6.3 software, and data collected for each condition were normalized with total protein values in each VAT fragment.

#### Protein extraction and quantification

After the bioenergetic assays, VAT fragments were used for total protein extraction to normalize OCR values to the protein content in each well. Protein isolation was performed following a protocol optimized for AT [19]. VAT fragments were incubated in RIPA buffer (RO278-500 ML, Sigma-Aldrich), supplemented with protease (04693124001, Roche, Basel, Switzerland) and phosphatase inhibitors (04906845001, Roche, Switzerland), overnight at 4°C. The day after, VAT fragments were lysed in a FastPrep®24 homogenizer (MP Biomedicals, Irvine, CA, USA), operating at 6.0 m/s for 30 seconds, three times, intercalated with 5-minute rest intermissions on ice. The homogenates were then incubated on ice for 1 hour and later centrifuged at 20,000 g for 15 minutes at 4°C.

For protein quantification, the commercial Pierce BCA Protein Assay Kit (23225, Thermo Fisher Scientific, Rockford, USA) was employed. Bovine Serum Albumin (BSA) standards, ranging from 0 to 2000 µg/mL, and the working reagent were prepared according to the manufacturer’s protocol. A 96-well microplate was loaded, in duplicate, with 25 µL of either sample or BSA standard, followed by the addition of 200 µL of working reagent. The plate was then incubated at 60°C for 30 minutes in the dark. Subsequently, the absorbance was read at 570 nm, in a microplate reader (Thermo Scientific™ Multiskan™ FC Microplate Photometer, Thermo Fisher Scientific, Rockford, IL, USA).

#### Bioenergetic parameters calculations

Basal oxygen consumption, acute responses to substrate deprivation, ATP-linked respiration, proton leak, non-mitochondrial respiration, maximal respiration, and spare respiratory capacity in VAT were measured as previously described [18] and represented in Figure 1.

**Figure 1.**
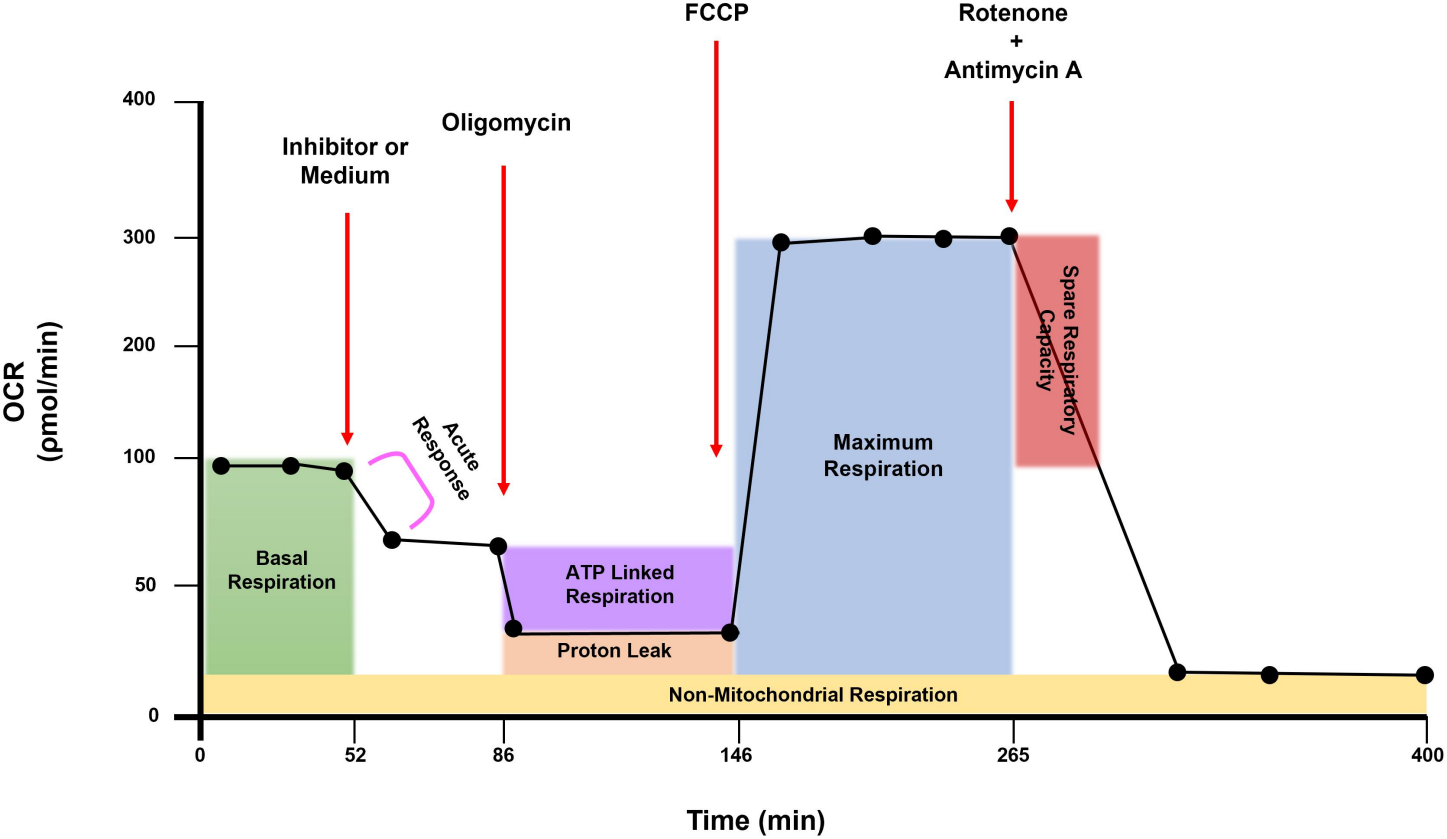
Schematic representation of the Seahorse assay and respective respiratory parameters calculations. Adapted from [20]. ATP: adenosine triphosphate; ETC: electron transport chain.

### Gene expression analysis

#### RNA isolation and quantification

Total RNA was extracted from the VAT using the RNeasy Lipid Tissue Kit (74804, QIAGEN, Germany), according to the manufacturer’s instructions. The RNA was quantified using a NanoDrop One spectrophotometer (Thermo Scientific, Thermo Fisher Scientific, USA).

#### cDNA synthesis

The Maxima First Strand cDNA Synthesis Kit (K1671, Thermo Fisher Scientific Inc, USA) was used in this assay to synthesize cDNA. For this, 250ng of RNA were incubated with Double-Strand Specific DNases for 2 minutes in order to eliminate gDNA. Afterwards, 10 µL each of the 5x Reaction Mix and the Maxima Enzyme Mix were added. In a T100TM Thermal Cycler (Bio-Rad, USA), the samples were then incubated using the following protocol: 10 minutes at 25°C, followed by 15 minutes at 50°C, and, lastly, 5 minutes at 85°C. Once again, these samples were stored at −80°C.

#### Quantitative real-time polymerase chain reaction analysis

Gene expression analysis of *OB* (leptin), *CPT1a* (Carnitine Palmitoyl Transferase 1a), *GLS1*, *MPC1* (Mitochondrial Pyruvate Carrier 1), *PDHA1* (Pyruvate Dehydrogenase A1) and *HK2* (Hexokinase 2), as well as the housekeeping genes *β-actin* and *RPII* (RNA polymerase II), was performed using previously validated primers (Table 1) and quantitative real-time PCR (qRT-PCR) with the NZYSpeedy qPCR Green Master Mix (2) (MB22402, NZYTech, Portugal). The housekeeping genes were selected based on prior validation studies involving human VAT [21]. Negative controls lacking cDNA were included, and all reactions were run in duplicate. Following mixture preparation, samples were loaded into the CFX Connect™ Real-Time PCR System (Bio-Rad, USA). Data analysis was conducted using the CFX Maestro™ software (Bio-Rad). The comparative CT method was used to analyse the qRT-PCR results. The following formula was used for calculating individual relative gene expression values: *Fold change = 2^–∆CT^ = 2 ^– (CT gene of interest^ ^– CT housekeeping gene)^* [22].

**Table 1.** Oligonucleotides and cycling conditions for PCR amplifications.

Gene	Primers sequence (F/R)	T_Annealing_ (ºC)	Size (bp)
*OB*	TCTTGTCCCCTCTTGACCCA AGCTTGTGTTGCTGGGAGTT	56	183
*CPT1a*	GCAGCGTTCTTTGTGACGTT AGGAGTGTTCAGCGTTGAGG	56	184
*GLS1*	TCTACAGGATTGCGAACGTCT CTTTGTCTAGCATGACACCATCT	56	100
*MCP1*	ATTTGCCTACAAGGTACAGCC AGTCATCTCGTGTTTGATAAGCC	56	109
*PDHA1*	CAGACTGTACGCCGAATGGA CCTGACCATCACACAAGTGAC	58	88
*HK2*	CGAGAGCATCCTCCTCAAGTG AGCCACAGGTCATCATAGTTCC	58	164
*RPII*	CTTCACGGTGCTGGGCATT GTGCGGCTGCTTCCATAA	50	239
*β-actin*	CCAACCGCGAGAAGATGA CCAGAGGCGTACAGGGATAG	50	97

Abbreviations: OB – leptin; CPT1a – Carnitine Palmitoyl Transferase 1a; GLS1 – glutaminase 1; MPC1 - Mitochondrial Pyruvate Carrier 1; PDHA1 - Pyruvate Dehydrogenase A1; HK2 - Hexokinase 2; RPII – RNA polymerase II.

#### Statistical analysis

GraphPad® Prism software (USA), version 8.0.1 for Windows, was used for the statistical analysis. Qualitative and Quantitative variables are expressed as percentages (%) and mean ± standard error of the mean, accordingly. Comparisons among the three glycaemic groups (euglycemia, prediabetes, and type 2 diabetes) were performed using one-way analysis of variance (ANOVA) for normally distributed variables and the Kruskal–Wallis test for non-normally distributed variables. Post hoc multiple-comparison tests were subsequently performed using Tukey’s test following one-way ANOVA and Dunn’s test following the Kruskal–Wallis test. For comparisons among the different inhibition conditions within the same VAT samples, the Friedman test followed by Dunn’s multiple comparisons test was used, with each inhibitor condition compared against the vehicle condition. A *p* < 0.05 was considered statistically significant.

## Results

### Participants’ characteristics

The characteristics of the individuals who participated in this study are presented in Table 2. No statistically significant differences were observed between groups in age, BMI, dyslipidemia prevalence, or the indirect markers of systemic inflammation assessed, namely the neutrophil-to-lymphocyte ratio (NLR) and platelet-to-lymphocyte ratio (PLR). Fasting plasma glucose was significantly higher in individuals with T2D than in those with euglycemia, while HbA1c was significantly higher in individuals with T2D than in those with euglycemia or prediabetes. None of the participants had a formal diagnosis of hepatic steatosis or MASLD. Among the individuals with dysglycemia, 5 individuals with T2D and 2 with prediabetes were under treatment with metformin and 1 patient with T2D was under gliclazide treatment. As per standard of care and for clinical safety reasons, metformin was withdrawn the day before surgery to minimize the risk of lactic acidosis driven by mitochondrial respiratory complex I inhibition and tissue hypoperfusion, allowing a minimum of 12 h of pharmacological washout.

**Table 2.** Anthropometric characteristics of this study participants.

	Total	Euglycemia	Prediabetes	T2D	*p* value
No. patients	29	11	12	6	–
Age (years)	47.00 ± 16.25	39.36 ± 12.61	50.55 ± 8.91	48.33 ± 2.50	NS
BMI (kg/m^2^)	45.28 ± 5.12	42.93 ± 5.09	48.67 ± 6.07	43.55 ± 5.19	NS
HbA1c (%)	5.58 ± 0.38	5.31 ± 0.18	5.86 ± 0.30	6.93 ± 1.19	*p < 0.05 #*p* < 0.001
FPG (mg/dL)	98.00 ± 15.32	89.78 ± 11.73	107.25 ± 13.94	133.00 ± 35.08	Ւ*p* < 0.01
Dislipidemia (%)	10 (34.48)	5 (45.45)	3 (25.00)	2 (33.33)	NS
NLR	2.19 ± 0.27	1.98 ± 0.23	2.69 ± 0.75	1.88 ± 0.28	NS
PLR	113.60 ± 9.20	97.64 ± 8.46	126.30 ± 21.44	123.30 ± 18.40	NS

*euglycemia vs prediabetes, # euglycemia vs T2D, Ւ euglycemia vs T2D. BMI: Body Mass Index; FPG: fasting plasma glucose; HbA1c: glycated haemoglobin; T2D: Type 2 Diabetes; NLR: neutrophil-to-lymphocyte ratio; PLR: platelet-to-lymphocyte ratio.

### VAT basal oxygen consumption rate was similar across glycaemic statuses

Under basal conditions, i.e. without inhibition of any metabolic pathway substrates, no significant differences were observed in the VAT respiratory parameters across the different glycaemic statuses (Figure 2).

**Figure 2.**
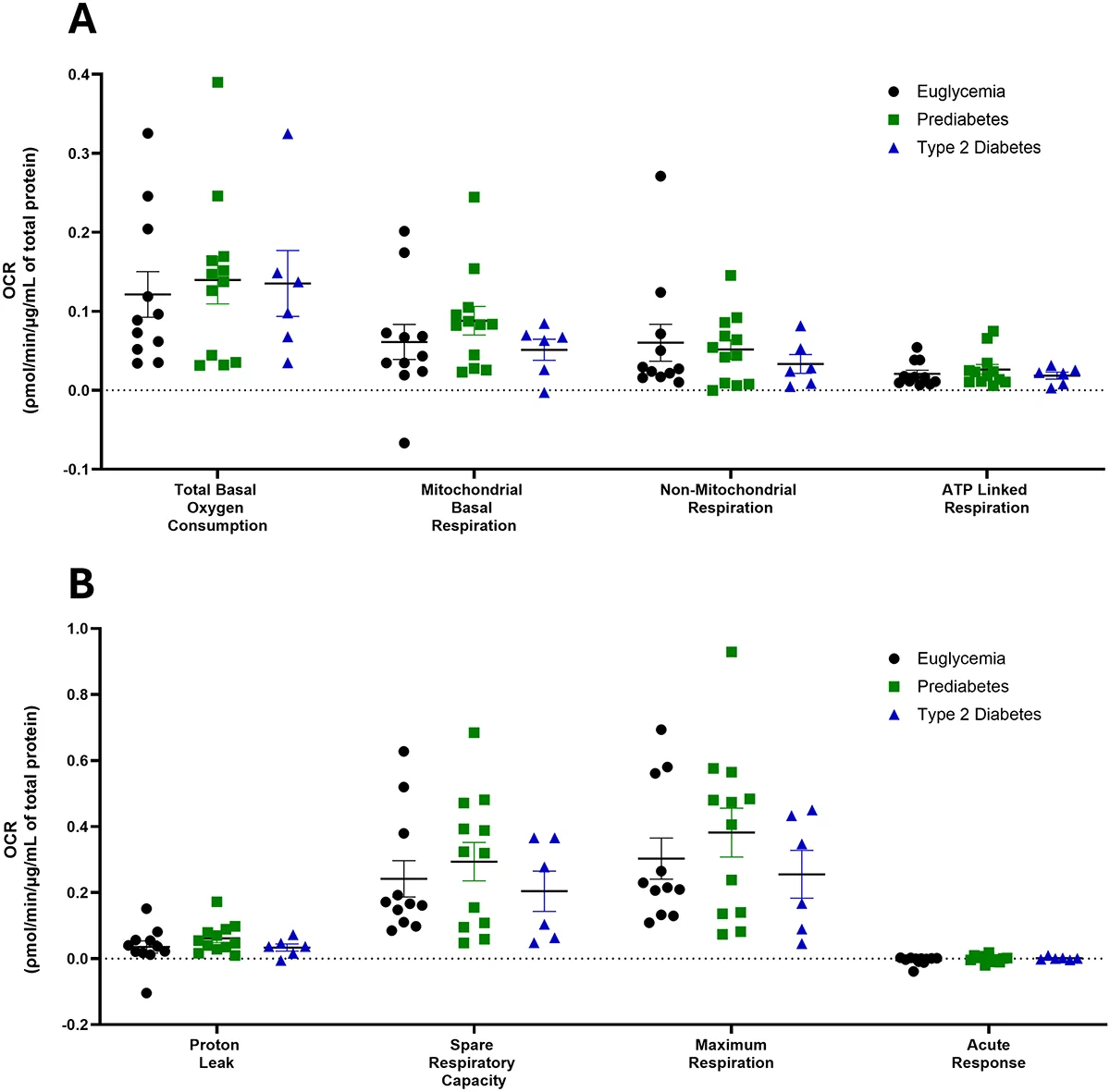
Respiratory parameters evaluated by oxygen consumption rate (OCR) within visceral adipose tissue (VAT) of individuals with euglycemia, prediabetes or type 2 diabetes analysed.

### VAT responds differently to substrate oxidation pathways inhibition depending on glycemic status

#### Euglycemia

In the euglycemic group, the only respiratory parameter that showed significant changes in response to the metabolic inhibitors was the spare respiratory capacity (Figure 3). This parameter was significantly reduced when VAT was exposed to UK5099 alone (100.00 ± 21.52% vs. 36.04 ± 5.70%, *p* < 0.05), or in combination with Etomoxir (100.00 ± 21.52% vs. 24.30 ± 6.41%, *p* < 0.01) or BPTES (100.00 ± 21.52% vs. 30.36 ± 6.99%, *p* < 0.01), compared to the control condition (i.e. without any inhibition) (Figure 3(F)).

**Figure 3.**
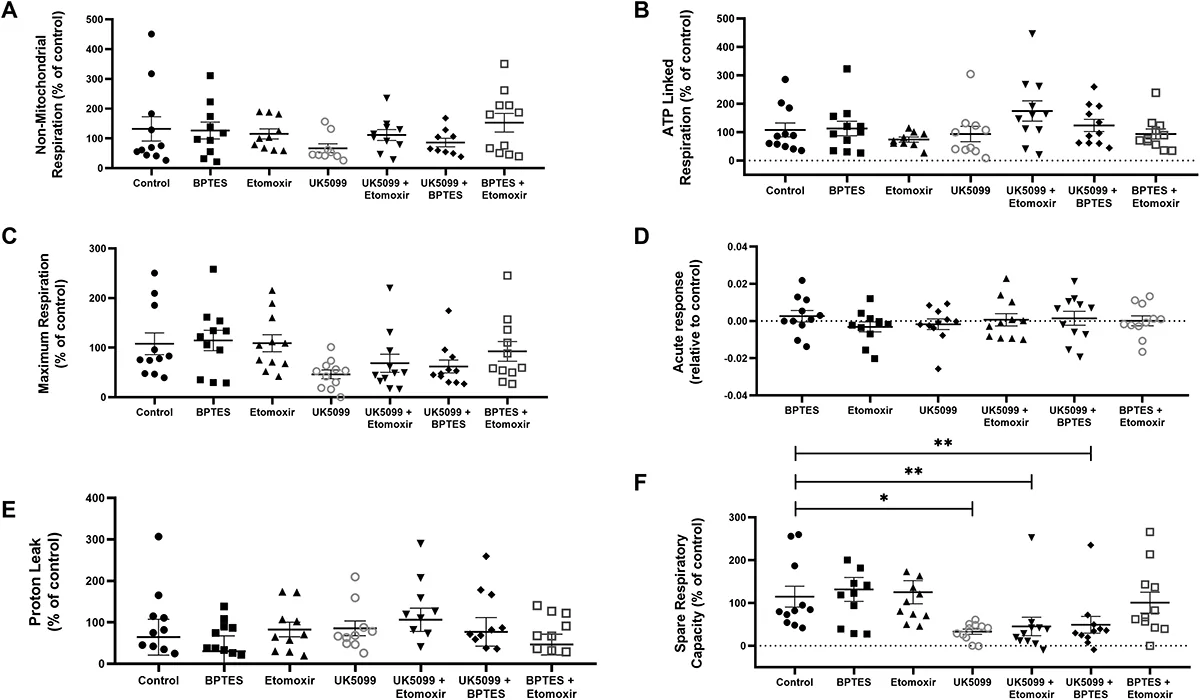
Respiratory parameters under the influence of specific substrate oxidation pathways inhibitors (BPTES, Etomoxir, UK5099) for visceral adipose tissue (VAT) from individuals with euglycemia: (A) Non-mitochondrial respiration; (B) ATP-Linked respiration; (C) Maximum respiration; (D) Acute response; (E) Proton Leak; (F) Spare respiratory capacity. Data are expressed as mean ± SEM (*n* = 11). Statistical significance was determined as *p < 0.05; **p < 0.01. BPTES - ((bis-2-(5-phenylacetamido-1,3,4-thiadiazol-2-yl)ethyl sulphide) glutaminase inhibitor; Etomoxir – carnitine palmitoyltransferase inhibitor; UK5099 – mitochondrial pyruvate carrier 1 inhibitor.

#### Prediabetes

In prediabetes, VAT non-mitochondrial respiration and ATP-linked respiration increased significantly when exposed to the combination of UK5099 and Etomoxir (100.00 ± 34.35% vs. 184.40 ± 30.05%, *p* < 0.01; 100.00 ± 21.72% vs. 231.7 ± 43.71%, *p* < 0.05, respectively), when compared with the control condition (i.e. without inhibition) (Figure 4(A,B)). No other significant differences were observed (Figure 4).

**Figure 4.**
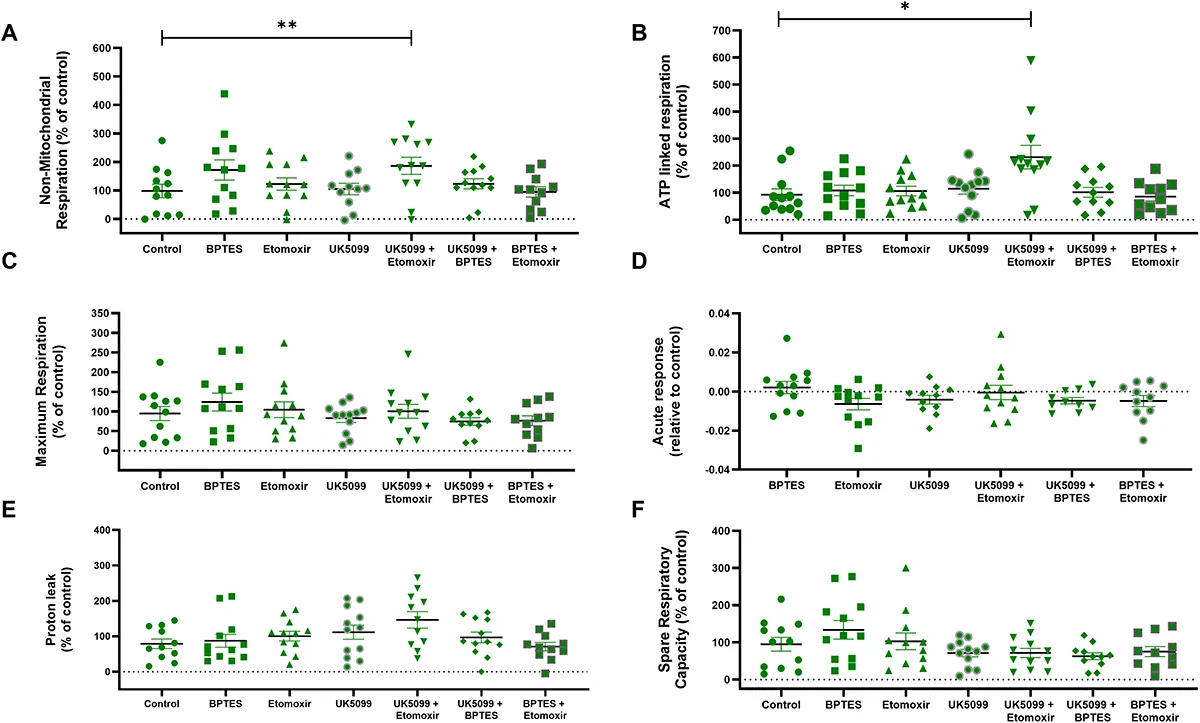
Respiratory parameters under the influence of specific substrate oxidation pathways inhibitors (BPTES, Etomoxir, UK5099) for visceral adipose tissue (VAT) from individuals with prediabetes: (A) Non-mitochondrial respiration; (B) ATP-Linked respiration; (C) Proton Leak; (D) Spare respiratory capacity; (E) Maximum respiration; (F) Acute response. Data are expressed as mean ± SEM (*n* = 12). Statistical significance was determined as *p < 0.05; **p < 0.01. BPTES - ((bis-2-(5-phenylacetamido-1,3,4-thiadiazol-2-yl)ethyl sulphide) glutaminase inhibitor; Etomoxir – carnitine palmitoyltransferase inhibitor; UK5099 – mitochondrial pyruvate carrier 1 inhibitor.

#### Type 2 diabetes

In T2D, VAT ATP-linked respiration increased significantly under combined inhibition by UK5099 and Etomoxir (100.00 ± 28.02% vs. 193.3 ± 70.81%, *p* < 0.05) (Figure 5(B)). No other significant differences were observed (Figure 5).

**Figure 5.**
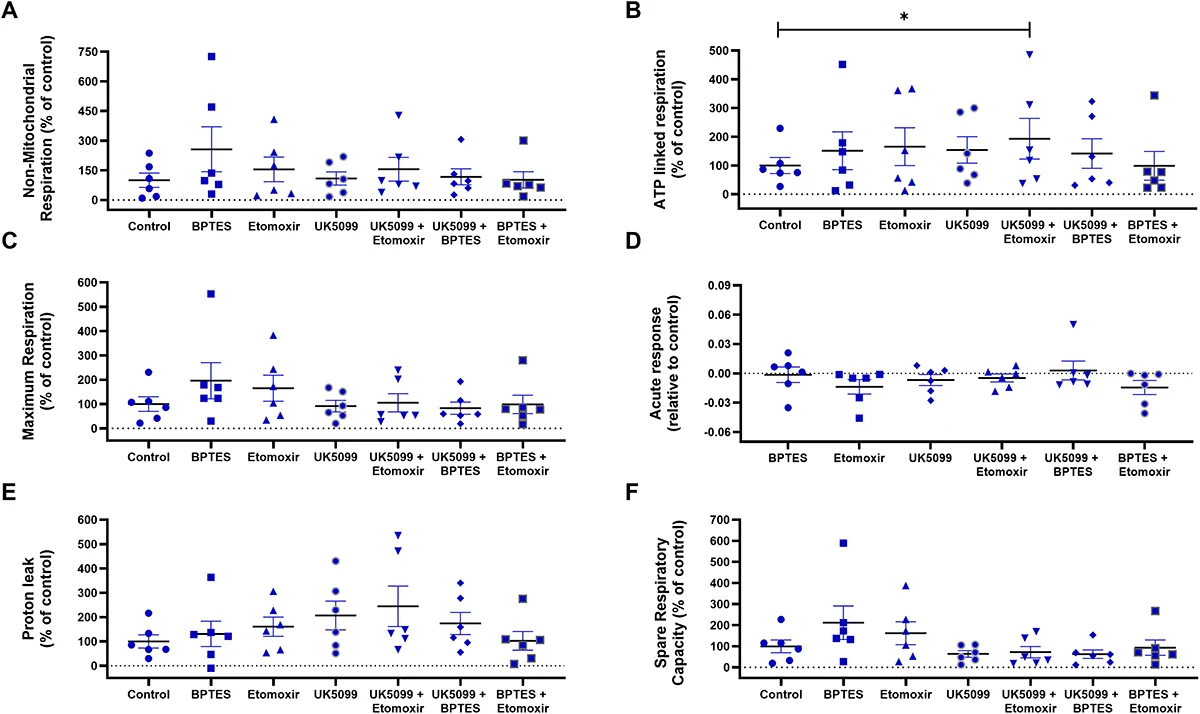
Respiratory parameters under the influence of specific substrate oxidation pathways inhibitors (BPTES, Etomoxir, UK5099) for visceral adipose tissue (VAT) from individuals with type 2 diabetes (T2D). (A) Non-mitochondrial respiration; (B) ATP-Linked respiration; (C) Proton Leak; (D) Spare respiratory capacity; (E) Maximum respiration; (F) Acute response. Data are expressed as mean ± SEM (*n* = 6). Statistical significance was determined as *p < 0.05; **p < 0.01. BPTES - ((bis-2-(5-phenylacetamido-1,3,4-thiadiazol-2-yl)ethyl sulphide) glutaminase inhibitor; Etomoxir – carnitine palmitoyltransferase inhibitor; UK5099 – mitochondrial pyruvate carrier 1 inhibitor.

### Differences in mitochondrial function are not associated with different expression of genes involved in energy metabolism

VAT gene expression of *OB*, *CPT1a*, *MPC1*, *GLS1*, *PDHA1*, *HK2 and* the housekeeping genes – *RPII* and *β-actin* was assessed by qRT-PCR. This analysis showed no statistically significant differences among the glycaemic groups (Figure 6).

**Figure 6.**
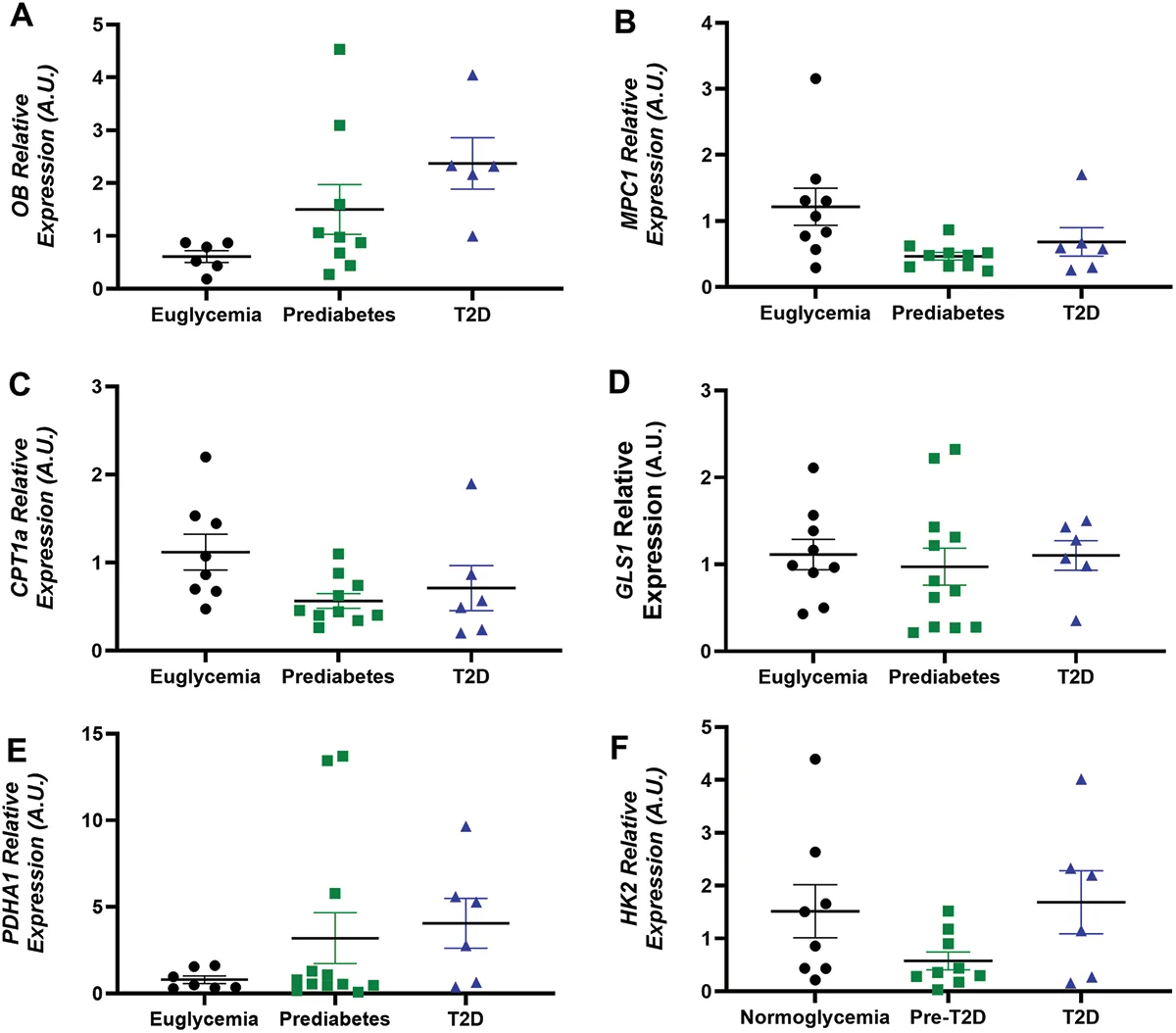
Gene relative expression housekeeping of (A) OB – leptin; (B) MPC1 – mitochondrial pyruvate carrier 1; (C) CPT1a – carnitine palmitoyltransferase 1a; (D) GLS1 – glutaminase 1; (E) PDHA1 – pyruvate dehydrogenase A1; (F) HK2 – Hexokinase 2. Results were normalized for the expression of two housekeeping genes (β-actin and RPII).

## Discussion

Uncovering tissue bioenergetics is crucial for understanding the energy flow within living systems. It plays a vital role in recognizing the pathological mechanisms responsible for energy imbalance disorders, identifying disease progression biomarkers, and pinpointing potential targets for intervention, setting the grounds for the development of innovative therapeutic approaches [23]. Previous studies showed that mitochondrial bioenergetics are altered in obesity and T2D [17,24]. However, whether VAT bioenergetics and substrate preferences in obesity differ across distinct glycaemic profiles remains incompletely understood. Therefore, the present study aimed to assess substrate utilization and metabolic flexibility in VAT from individuals with obesity stratified according to glycaemic status, namely euglycemia, prediabetes, and T2D.

In this study, we found that before any inhibition occurred, there were no significant differences in respiratory parameters of VAT from individuals with prediabetes or T2D when compared with those with euglycemia. This finding may be explained by the fact that, although prediabetes is characterized by early metabolic disturbances, it often represents a potentially reversible state where mitochondrial dysfunction may not yet be fully established or may remain functionally compensated [15,25,26]. Therefore, the similarities observed in mitochondrial respiration, in this group, may reflect the early stage of disease progression towards diabetes, where bioenergetic alterations are not yet evident. Additionally, metformin treatment, reported in five of the six individuals with T2D and two individuals with prediabetes, may have influenced the results. Metformin is a mitochondrial modulator that has been reported to improve mitochondrial fission and respiration in experimental settings [27], raising the possibility that treatment contributed to the absence of detectable basal respiratory differences. Conversely, although metformin can inhibit mitochondrial complex I, therapeutic exposure does not necessarily translate into reduced respiratory capacity measured *ex* *vivo*. Larsen et al. reported comparable complex I-supported and combined complex I- and II-supported respiration in skeletal muscle from metformin-treated individuals with T2D, sulfonylurea-treated individuals with T2D, and healthy controls [28]. These findings argue against assuming that metformin treatment necessarily suppresses *ex* *vivo* mitochondrial respiration. Nevertheless, these findings cannot be directly extrapolated from skeletal muscle to intact VAT, and treatment-related effects cannot be excluded in our cohort. The absence of significant differences should therefore be interpreted within the treatment context, rather than as evidence of preserved mitochondrial function independently of medication exposure. These findings should also be considered alongside studies relating VAT mitochondrial respiration to tissue-specific insulin sensitivity and hepatic metabolic status. Pafili et al. reported lower maximal mitochondrial respiration in VAT, but not SAT, in individuals with obesity and metabolic dysfunction-associated steatohepatitis compared with those without fatty liver disease [29]. Notably, lower VAT respiration was associated with lower AT insulin sensitivity, suggesting a relationship between VAT oxidative capacity and local insulin responsiveness. This association provides a relevant context for our findings, although classification by glycaemic status does not directly capture AT insulin sensitivity. In another study, Pedersen et al. found reduced oxidative phosphorylation capacity in VAT and SAT in individuals with obesity compared with controls without obesity, without an overall association with fatty liver severity [30]. Together, these studies highlight the importance of metabolic characterization, comparison groups, and respiratory endpoints when interpreting adipose tissue bioenergetics. Our study examined substrate-dependent respiratory responses across glycaemic states within a population with obesity. Therefore, the absence of significant differences in basal respiration does not exclude bioenergetic abnormalities shared across these groups or a relationship between VAT respiratory capacity and tissue-specific insulin resistance. Differences in cohort classification and respirometry protocols should also be considered when comparing these findings.

Our research also showed that VAT basal respiration is not influenced by the deprivation of any of the substrates, suggesting that VAT has a high metabolic flexibility to utilize all substrates as available. However, whenever energy demands increase, the preferred substrate for mitochondrial respiration in the VAT of individuals with euglycemia seems to be pyruvate, as demonstrated by the significant decrease in the spare respiratory capacity following inhibition of the mitochondrial pyruvate carrier. This finding is consistent with previous studies that have shown that adipocytes preferentially maintain pyruvate oxidation to preserve mitochondrial respiratory capacity, thus preventing an ATP crisis [31,32]. In addition, a previous study also showed that pyruvate is the preferential VAT substrate when energy is demanded [18]. In contrast, the spare respiratory capacity of VAT from individuals with prediabetes and T2D was not significantly affected by pyruvate deprivation. This divergent response indicates a fundamental shift in mitochondrial substrate preference in the presence of dysglycemia. In fact, recent evidence revealed that VAT from insulin-resistant individuals and those with T2D display markedly different substrate utilization patterns when compared to insulin-sensitive controls. Specifically, while VAT from insulin-sensitive individuals preferentially oxidizes pyruvate, malate, and glutamate during oxidative phosphorylation, VAT from individuals with T2D exhibits a notable shift towards succinate oxidation, reflecting altered TCA cycle substrate entry [17]. This metabolic reprogramming may represent an adaptive response to chronic hyperglycaemia, insulin resistance, and impaired mitochondrial coupling efficiency that characterizes T2D [17,33]. By shifting away from pyruvate-dependent pathways, mitochondria may avoid the defects in glucose utilization of VAT from individuals with dysglycemia.

In contrast, in the VAT of individuals with prediabetes, our results show a significant increase in oxygen consumption associated with ATP production when glutamine was the only available substrate. Glutamine is a non-essential amino acid used to produce energy through glutaminolysis. Glutamine is transported to the mitochondria and then converted into glutamate by glutaminase 1. This glutamate is later converted into the TCA cycle intermediate α-ketoglutarate by glutamate dehydrogenase [34,35]. Petrus et al. reported that glutamine can decrease the cell dependency on glycolysis by increasing the usage of glutamine in the TCA cycle activity for the production of NADH and FADH_2_, which serve as electron carriers in the ETC [36]. These pathways provide a possible explanation for the higher ATP-linked respiration observed in our study and suggest an adaptation towards greater reliance on glutamine to support oxidative phosphorylation. This metabolic response could represent an early mechanism of mitochondrial compensation when glucose utilization is restricted. However, mitochondrial oxidative metabolism can also generate ROS through electron leakage from the ETC, although increased oxidative phosphorylation does not necessarily imply greater ROS production [37,38]. If ROS production exceeds antioxidant capacity, the resulting oxidative stress may potentiate insulin resistance and promote metabolic dysfunction [39–41].

In parallel, non-mitochondrial oxygen consumption was also significantly increased under the same glutamine-alone status. This may reflect increased activity of oxygen-consuming enzymes, potentially including NADPH oxidases, which generate ROS involved in cellular signalling and immune responses [42,43]. The simultaneous rise in ATP-linked respiration and non-mitochondrial oxygen consumption raises the possibility that VAT from individuals with prediabetes engages both mitochondrial energy production and additional redox-related processes in response to restricted substrate availability. This pattern could represent an early metabolic adaptation, with glutamine supporting oxidative energy metabolism while accompanying changes in ROS-generating pathways influence cellular signalling and redox balance. However, the contribution of NADPH oxidases was not directly assessed, and increased non-mitochondrial oxygen consumption cannot be equated with enhanced antioxidant defence. Whether the observed response contributes to adaptive redox signalling or promotes oxidative stress remains unresolved. Thus, the proposed compensatory role of glutamine remains a hypothesis requiring direct measurements of ROS, antioxidant capacity, and the oxygen-consuming pathways involved. However, the limited amount of VAT available per individual precluded complementary experiments to quantify ROS production, assess antioxidant capacity, and investigate the contribution of specific oxygen-consuming pathways, including NADPH oxidases. Consequently, increased non-mitochondrial oxygen consumption cannot be equated with enhanced antioxidant defence, and whether this response contributes to adaptive redox signalling or promotes oxidative stress remains unresolved. The proposed compensatory role of glutamine should therefore be regarded as a mechanistic hypothesis requiring experimental confirmation.

Of particular note, VAT of individuals with T2D also presented higher OCR consumption related to ATP production when glutamine was the only substrate available. Previous studies reported altered glutamine metabolism in individuals with T2D, including lower glutamine and α-ketoglutarate concentrations in serum [44], and that glutamine may modulate VAT inflammatory status [45]. Interestingly, activated immune cells, for instance, macrophages and lymphocytes, shift their metabolism towards increased glutamine usage, making it a preferred substrate to sustain cell functions, namely proliferation and cytokine production [46]. Thus, differences in the abundance or metabolic activity of immune cells may contribute to the glutamine-associated respiratory profile of intact VAT, alongside contributions from adipocytes and other resident cells.

In contrast to the pattern observed in prediabetes, the increased ATP-linked mitochondrial respiration in VAT from individuals with T2D was not accompanied by a corresponding increase in non-mitochondrial oxygen consumption. This pattern may suggest reduced metabolic flexibility and compensatory capacity in T2D, potentially compromising redox regulation despite increased glutamine utilization. However, this interpretation remains hypothetical because redox balance and antioxidant capacity were not directly assessed. Metformin treatment in five of the six individuals with T2D should also be considered when interpreting this response. The higher ATP-linked respiration under the glutamine-only condition is not readily explained by a simple inhibitory effect of metformin on mitochondrial complex I, which, if sufficiently pronounced, would be expected to constrain NADH-linked oxidative respiration rather than enhance it. Nevertheless, this mechanistic expectation does not exclude treatment-related adaptations, as the corresponding response without metformin is unknown. The observed pattern therefore cannot be attributed specifically to T2D independently of treatment.

Future investigations should identify the pathways contributing to non-mitochondrial oxygen consumption, including NADPH oxidase activity, alongside direct measurements of oxidative stress and antioxidant capacity. Complementary assessment of mitochondrial uncoupling and the influence of metformin would help determine whether the different respiratory patterns observed in prediabetes and T2D reflect altered compensatory mechanisms, treatment-related effects, or both.

Metabolic flexibility is described as being determined by the interchange between metabolic pathways, which depend on gene and protein expression, nutrient availability, and metabolic demand [47]. In this study, no significant differences between the groups were observed in the expression of the analysed genes involved in energy metabolism and bioenergetics. These findings suggest that the observed bioenergetic differences are not accompanied by detectable changes in the expression of the genes examined. This may reflect regulation at levels beyond transcript abundance, including protein abundance, enzyme activity, or post-translational modifications, although these possibilities were not directly assessed. The results therefore raise the possibility that dysglycemia-associated changes in VAT bioenergetics involve altered engagement of substrate pathways rather than a generalized loss of oxidative capacity. However, the limited transcript panel does not allow broader transcriptional changes to be excluded.

This study has limitations that should be considered. The cross-sectional design prevents us from establishing causality or tracking the longitudinal trajectory of metabolic reprogramming from euglycemia through prediabetes to T2D. The availability of metabolic measurements across the cohort also limited the assessment of insulin resistance, hepatic fat content, and body-fat distribution, which could help further explain individual variation in VAT respiratory responses.

The relatively small sample size, particularly the T2D group comprising six individuals, limits statistical power to detect between-group differences. Nevertheless, the study provides exploratory functional data on substrate-dependent respiratory responses in human VAT across glycaemic states, which warrant confirmation in larger cohorts.

The limited volume of VAT available per patient restricted our ability to perform complementary subanalyses that could further elucidate the signalling pathways underlying the observed bioenergetic alterations, particularly those related to oxidative stress. Additionally, the use of intact VAT for bioenergetic assessment was consistent with our objective of characterizing substrate-dependent respiratory responses at the tissue level. This approach was chosen to retain tissue architecture and interactions among resident cell populations. Accordingly, the measured OCR reflects the integrated contribution of adipocytes and stromal vascular cells, including immune cells. Differences in cellular composition or activation state may therefore contribute to the observed respiratory profiles, and this approach cannot distinguish these contributions from metabolic changes within individual cell populations. To minimize the potential confounding effect of differences in adipocyte cell abundance per volume across the study groups, we analysed *OB* gene expression. The absence of significant differences in *OB* expression between groups supports the interpretation that the bioenergetic alterations identified are not merely a consequence of the different number of adipocytes, but rather reflect intrinsic metabolic shifts within VAT. Nevertheless, this finding does not establish comparable exclude differences in immune cell infiltration.

The VAT explants remained responsive to the sequentially administered compounds throughout the assay, providing functional evidence of maintained respiratory activity. A similar tissue mass of approximately 5 mg per well was used across all groups under the same assay conditions to reduce technical variability associated with sample loading. Although this standardization does not exclude potential oxygen diffusion effects within intact fragments, it reduces variation attributable to differences in the amount of tissue analysed. Furthermore, OCR was expressed per unit of total VAT protein, a normalization approach that does not fully account for potential differences in adipocyte size and tissue composition.

The potential confounding effect of antidiabetic therapies in the T2D group should also be taken into account when interpreting the results, as these drugs may influence metabolic and mitochondrial parameters. Nonetheless, this represents an inherent and unavoidable limitation, since withholding antidiabetic treatment for the purpose of methodological control would be ethically unacceptable.

In conclusion, this study found no statistically significant differences in basal VAT respiratory parameters across the spectrum of dysglycemia. Substrate-dependent respiratory responses nevertheless suggest that VAT bioenergetic adaptations may differ across the spectrum of dysglycemia. Under glutamine-only conditions, individuals with prediabetes exhibited increases in both ATP-linked mitochondrial respiration and non-mitochondrial oxygen consumption, whereas individuals with T2D exhibited an increase only in ATP-linked respiration. This pattern raises the possibility of altered compensatory responses in T2D, although the underlying mechanisms remain to be established. The absence of detectable differences in the transcripts examined suggests that regulation beyond transcript abundance, potentially including post-translational mechanisms, may contribute to these responses. These findings support further investigation of protein activity and signalling pathways as potential contributors to VAT metabolic adaptation, while requiring confirmation in larger cohorts.

## Data Availability

The participants of this study did not give written consent for their data to be shared publicly, so due to the sensitive nature of the research, supporting data is not available.
